# Dietary Dried Laver (Porphyra tenera) Modulates Gut Microbiota Composition and Diversity in Older Women with and Without Metabolic Syndrome: An Exploratory Pilot Study

**DOI:** 10.3390/nu18101535

**Published:** 2026-05-12

**Authors:** Dayeon Shin, Suyeon Lee, Byunghun So, Chounghun Kang, Kyung Ju Lee

**Affiliations:** 1 Department of Nutritional Science and Food Management, College of Science and Industry Convergence, Ewha Womans University, Seoul 03760, Republic of Korea; 2 Department of Food and Nutrition, College of Natural Sciences, Inha University, Incheon 22212, Republic of Korea; 3 Department of Health and Exercise Science, College of Graduate School, Inha University, Incheon 22212, Republic of Korea; 4 Department of Physical Education, College of Education, Inha University, Incheon 22212, Republic of Korea; 5 Dongjak-gu Public Health Center, Seoul 06925, Republic of Korea

**Keywords:** *Porphyra tenera*, gut microbiota, metabolic syndrome, alpha diversity, short-chain fatty acids (SCFAs)

## Abstract

**Background:** Metabolic syndrome (MetS) is a cluster of cardiometabolic abnormalities linked to increased risk of type 2 diabetes and cardiovascular disease. Emerging evidence implicates gut microbiota dysbiosis in MetS pathophysiology; however, human clinical data on seaweed-based dietary interventions remain scarce. **Methods:** Twenty-four older women were stratified into a MetS group (n = 13) and a control group (n = 11) per NCEP-ATP III criteria with Korean-specific waist circumference cutoffs. All participants consumed 4 g of dried laver (*Porphyra tenera*) per day for 12 weeks. Fecal DNA was subjected to 16S rRNA gene amplicon sequencing (V4 region; Illumina iSeq 100). Bioinformatic processing used QIIME2 and MicrobiomeAnalyst; alpha diversity was quantified by Chao1 and Fisher indices; beta diversity by Bray–Curtis, Jensen–Shannon, and UniFrac metrics; and differential abundance by LEfSe. **Results:** The Firmicutes/Bacteroidetes ratio declined 0.81-fold in the control group and 0.54-fold in the MetS group. Alpha diversity (Chao1 and Fisher indices) increased significantly in both groups (*p* < 0.001 and *p* < 0.05, respectively). Unweighted UniFrac distance showed significant compositional differences (R^2^ = 0.141, *p* = 0.001). LEfSe identified four FDR-significant genera: CAG_873 in the control group and Muribaculaceae, *Paraprevotella*, and *Tyzzerella* in the MetS group. **Conclusions:** Twelve-week dried laver supplementation produced measurable shifts in gut microbial diversity and community composition in older women, with potentially greater responsiveness in those with MetS. These preliminary findings justify adequately powered randomized controlled trials to evaluate laver as a gut-microbiome-targeted dietary strategy for MetS.

## 1. Introduction

Metabolic syndrome (MetS) represents a clinically significant clustering of interrelated cardiometabolic abnormalities whose global prevalence has risen sharply over recent decades, increasing from an estimated 11.9% in 2000 to 28.4% in 2023 among adults [1]. Characterized by the concurrent presence of at least three of five recognized components—central adiposity, hypertriglyceridemia, reduced high-density lipoprotein (HDL) cholesterol, elevated blood pressure, and impaired fasting glucose—MetS markedly increases susceptibility to type 2 diabetes, cardiovascular disease, non-alcoholic steatohepatitis, and certain malignancies [2,3]. National surveillance data from the Korea National Health and Nutrition Examination Survey (KNHANES) indicate that the prevalence of MetS among the Korean population increased from 22.8% in 2007 to 28.6% in 2022, with considerable variation across age and sex groups [4]. Notably, women aged 65 years and older bear a disproportionately elevated burden relative to their male counterparts [5,6]. This sex-specific disparity is widely attributed to the postmenopausal decline in circulating estrogens and concomitant rise in androgen activity, which together promote visceral fat deposition and abdominal obesity [7]. Because the pathophysiological drivers of MetS differ meaningfully by sex and age, preventive and therapeutic strategies must be tailored accordingly—yet effective, targeted interventions for postmenopausal women remain limited.

Among the biological mechanisms through which modifiable lifestyle factors—particularly diet—shape MetS risk, the gut microbiome has emerged as a pivotal intermediary. The gut microbiome—a dense ecosystem of approximately 100 trillion bacteria, viruses, and yeasts that coexist in a symbiotic relationship with the human host—has emerged as a pivotal modulator of cardiometabolic health [8]. Disruption of normal microbiome composition, driven by factors such as poor dietary quality, physical inactivity, and antibiotic exposure, precipitates wide-ranging physiological disturbances that collectively elevate MetS risk [9]. Enterotyping of gut microbiome profiles in patients receiving MetS treatment revealed three compositionally distinct community types; among these, the enterotype characterized by high *Prevotella* 9 abundance was most strongly associated with modifiable behavioral exposures, including elevated smoking frequency and alcohol consumption [10]. Diet in particular exerts a dominant influence on microbiome structure: both acute dietary interventions and habitual long-term intake patterns alter species composition, with non-digestible plant cell wall polysaccharides emerging as especially potent determinants of beneficial microbial community membership [11,12].

Seaweed has constituted a dietary staple across East Asian cultures—including Korea, Japan, and China—for centuries, and interest in its health-promoting properties is now expanding rapidly in Western markets [13,14]. Among the diverse range of edible algae, laver (*Porphyra* spp.) is one of the most widely consumed, valued for its low caloric density and generous supply of carbohydrates, protein, vitamins, and minerals [15]. *Porphyra tenera* is further distinguished by bioactive constituents—including the sulfated polysaccharide porphyran, vitamin B12, and taurine—that are largely absent from other seaweed species and from terrestrial plant foods [16]. Marine algal polysaccharides resist enzymatic digestion in the proximal gastrointestinal tract, arriving intact in the colon where they can selectively modulate microbial community composition [17]. Preclinical evidence supports a role for *Porphyra*-derived bioactives in reshaping the gut microbiome: *Porphyra haitanensis* glycoproteins promoted the growth of Muribaculaceae and *Lachnospiraceae* while improving glucose metabolism in hyperglycemic mice [18], and porphyran from the same species significantly increased *Roseburia* and *Eubacterium* and suppressed *Helicobacter* abundance, with accompanying reductions in lipid accumulation, in obese mice [19]. *Porphyra tenera* itself has demonstrated immunomodulatory, antioxidant, and anti-inflammatory activities [15,20,21], and *Porphyra tenera* extract ameliorated colitis-associated inflammation in mice by favorably modulating intestinal microflora composition [22]. Nevertheless, the direct applicability of these findings to human health remains uncertain, given the pronounced interspecies differences in gut microbiome architecture and the widespread use of genetically modified rodent models that do not fully recapitulate human MetS [8].

Although the biological plausibility of laver as a microbiome-targeted dietary intervention for MetS is well supported by preclinical data, human clinical evidence addressing its effects on gut microbiota composition—particularly in older women at elevated MetS risk—is entirely lacking. Furthermore, individual variability in metabolic responses, shaped by diet, genetics, lifestyle, and baseline microbiome composition, warrants explicit consideration in this population [11]. The present exploratory pilot study was therefore conducted to investigate the effects of 12 weeks of daily dried laver (*Porphyra tenera*) consumption on gut microbiota diversity and abundance in older women, with pre-specified comparisons between those with and without MetS.

## 2. Methods

### 2.1. Study Material: Dried Laver

The dietary intervention material comprised commercially harvested dried laver of the species *Porphyra tenera*, a red macroalga belonging to the phylum Rhodophyta, class Rhodophyceae, order Bangiales, and family Bangiaceae. Raw laver was collected from the coastal waters of Jangheung, Jeollanam-do, Republic of Korea (34°36′10.4″ N, 127°05′52.7″ E) across the winter-to-spring harvesting window from December 2022 through March 2023. To isolate the intrinsic biological effects of laver from the confounding nutritional contributions of common condiments, the product was roasted in the absence of sesame oil and salt—ingredients standard in Korean culinary practice. The finished intervention material was portioned into individual sealed packs, each containing eight sheets (2 g).

### 2.2. Study Design and Participant Enrollment

**Figure 1:**
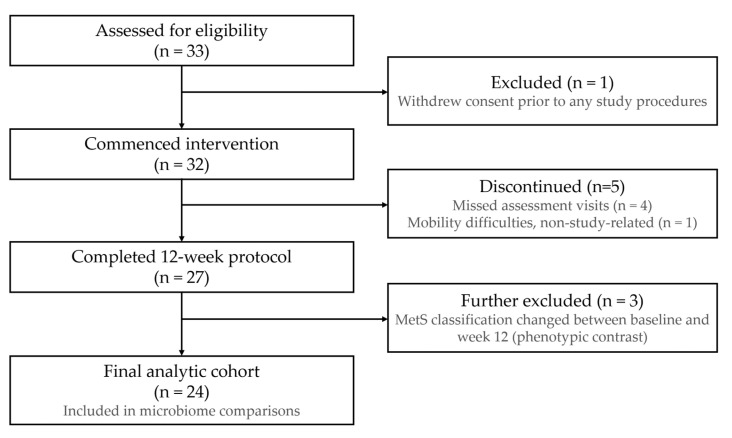
Flow chart of participants.

Community-dwelling adults and home care workers were invited to participate through recruitment notices distributed in cooperation with local home care facilities. Eligibility required that candidates be women aged 50 years or older, physically capable of incorporating dried laver into their habitual diet, and willing to complete all scheduled study visits and assessments. Before enrollment, all prospective participants received a detailed verbal and written explanation of study procedures, including the nature and duration of the intervention, and provided signed informed consent. An eligibility screening battery—comprising a structured health and lifestyle questionnaire, standardized physical examination, and fasting blood draw for biochemical profiling—was administered to 33 female volunteers. Those with significant mobility limitations or dependence in activities of daily living were excluded as ineligible. One volunteer withdrew consent before commencing any study procedures, leaving 32 participants who initiated the 12-week intervention. Of these, 27 completed the full protocol; five were excluded for protocol deviations (four failed to attend all assessment visits; one developed mobility difficulties of personal, non-study-related origin). Our clinical trial (Registration number: KCT0011947) was approved and registered with the Korea Disease Control and Prevention Agency—Clinical Research Information Service (CRIS) on 8 May 2026. To achieve a clean phenotypic contrast for microbiome comparisons, participants whose MetS classification changed between baseline and week 12 were additionally excluded, yielding a final analytic cohort of 24 women (Figure 1).

Field data collection took place between June and September 2023. All enrolled participants were instructed to consume one portion pack of dried laver at each of two main meals per day (two packs daily; 4 g/d total), corresponding to the single-serving recommendation of the Application of Dietary Reference Intakes for Koreans 2020. Adherence to laver intake was assessed through weekly telephone contact and self-reported consumed portion packs. A minimum adherence threshold of 80% was met by all 24 analytic participants. Baseline questionnaires on personal demographics and medical history were completed prior to the first assessment. The trial was conducted as an open-label, single-arm, repeated-measures panel study, with comprehensive assessments at week 0 (baseline) and week 12 (post-intervention). A study coordinator maintained regular telephone contact with participants to monitor intervention adherence, document health status changes, and record any adverse events. The study protocol received ethical approval from the Institutional Review Board of the National Rehabilitation Center on 24 April 2023 (IRB No. NRC-2023-03-019).

### 2.3. Metabolic Syndrome Classification

MetS was defined in accordance with the criteria of the National Cholesterol Education Program Expert Panel on Detection, Evaluation, and Treatment of High Blood Cholesterol in Adults (NCEP-ATP III) [23], with the waist circumference cutoff adapted to the sex-specific standards published by the Korean Society for the Study of Obesity [24]. A participant was classified as having MetS upon satisfying three or more of the following five criteria: (1) waist circumference of 85 cm or greater in women (90 cm or greater in men); (2) fasting serum triglycerides of 150 mg/dL or higher, or receipt of lipid-lowering pharmacotherapy; (3) HDL cholesterol below 50 mg/dL in women (below 40 mg/dL in men); (4) systolic blood pressure of 130 mmHg or higher, or diastolic blood pressure of 85 mmHg or higher, or current use of antihypertensive agents; and (5) fasting plasma glucose of 100 mg/dL or higher, or current use of glucose-lowering medication.

### 2.4. Anthropometric and Blood Pressure Measurements

BMI was computed as body weight in kilograms divided by the square of standing height in meters. Waist circumference was measured horizontally at the midpoint between the inferior margin of the lowest rib and the superior border of the iliac crest, to the nearest 0.1 cm; the mean of two consecutive readings served as the analytical value. Resting blood pressure was assessed with a validated automated digital sphygmomanometer (PG-800B11; Vitagram, Gyeonggi-do, Republic of Korea). The inflatable cuff was positioned over the brachial artery of the upper arm while the participant was seated quietly, and measurements were obtained bilaterally; the mean of the right- and left-arm readings was recorded.

### 2.5. Fecal Sample Collection and DNA Extraction

Prior to each collection point, participants attended a training session on standardized stool collection procedures. They were instructed to affix a collection sheet to the toilet seat and transfer approximately 2 g of freshly deposited feces into a pre-labeled sterile container. Collection was timed to occur one to three days before each scheduled visit; samples were stored immediately at −18 °C by the participant and transported to the laboratory on the assessment day, where they were archived at −80 °C until extraction.

For DNA extraction, frozen aliquots were thawed at 4 °C and 200 mg of each sample was processed with the SPINeasy DNA Kit for Feces (MP Biomedicals, Irvine, CA, USA) per the manufacturer’s protocol. Mechanical cell lysis was achieved with a FastPrep-24 5G bead homogenizer (MP Biomedicals) operated at 6.0 m/s for 40 s. Extracted DNA was visualized by 1% agarose gel electrophoresis (Mupid-One; Takara Bio, Shiga, Japan), and integrity was confirmed with a ChemiDoc imaging system (Bio-Rad, Hercules, CA, USA).

### 2.6. 16S rRNA Gene Amplicon Sequencing

The extracted DNA was amplified using amplicon sequencing targeting the V4 region of the 16S rRNA gene. We used V4 region-specific primers along with locus-specific overhang sequences for polymerase chain reaction (PCR). The sequences used were 515F-5′-TCGTCGGCAGCGTCAGATGTGTATAAGAGACAG-GTGCCAGCMGCCGCGGTAA-3′ and 806R-5′-GTCTCGTGGGCTCGGAGATGTGTATAAGAGA-CAGGGACTACHVGGGTWTCT-AAT-3′. PCR amplification was carried out in a T100 Thermal Cycler (Bio-Rad) using 25 μL reactions containing 5 ng template DNA, 1 μL each of forward and reverse primer, and 12 μL of 2× KAPA HiFi HotStart ReadyMix. Thermal cycling conditions comprised an initial denaturation at 95 °C for 3 min, followed by 25 cycles of 95 °C for 30 s, 55 °C for 30 s, and 72 °C for 30 s, with a terminal extension at 72 °C for 5 min and a final 4 °C hold. Dual indices and sequencing adapters were incorporated through a second PCR using the Nextera XT Index Kit V2 Set A (Illumina, San Diego, CA, USA) under the same cycling conditions reduced to 8 amplification cycles. Primer dimers and residual primers were removed by AMPure XP bead purification (Beckman Coulter, Inc., Brea, CA, USA). Library concentrations were determined with a Qubit 4 Fluorometer (Thermo Fisher Scientific, Inc., Waltham, MA, USA) using the dsDNA High-Sensitivity Assay Kit. Final sequencing was performed on an Illumina iSeq 100 system in 2 × 150 bp paired-end mode using the iSeq 100 i1 Reagent v2 (300-cycle) cartridge, with PhiX Control v3 (Illumina, Inc., San Diego, CA, USA) included as a sequencing quality control.

### 2.7. Bioinformatic and Statistical Analysis

Raw paired-end reads were processed within the QIIME2 platform (version 2023.07). The DADA2 plugin performed quality-based filtering, primer removal, per-sample error modeling, denoising, chimera detection and removal, singleton exclusion, read merging, and dereplication, generating a table of amplicon sequence variants (ASVs). Taxonomy was assigned to each ASV by querying the SILVA 138.1 reference database with a pre-trained Naive Bayes classifier at 99% sequence identity. ASV and taxonomy tables were exported in comma-separated value format and imported into the MicrobiomeAnalyst web platform (https://www.microbiomeanalyst.ca/) for downstream analysis. Sequencing generated a total of 1,712,847 reads across all samples (mean 35,684 reads per sample; range 20,884 to 65,282). No rarefaction was performed, because sequencing depth did not vary by more than 10-fold across samples. Instead, total sum scaling (TSS) was applied in MicrobiomeAnalyst, and no additional data transformation was performed. Features with fewer than four raw reads were filtered; a prevalence filter retained only features detected in at least 20% of samples; and a low-variance filter based on the 10th interquartile range removed an additional 29 features, leaving 256 features for all analyses. No explicit batch-correction procedure was applied, because all samples were sequenced in the same run. Alpha diversity was quantified using Chao1 (richness) and Fisher (evenness) indices; overall differences among the four study groups were assessed using Kruskal–Wallis tests, and post hoc pairwise comparisons were performed using Wilcoxon rank-sum tests with Benjamini–Hochberg false discovery rate (FDR) adjustment. Beta diversity was examined by principal coordinates analysis (PCoA) using Bray–Curtis dissimilarity, Jaccard distance, unweighted UniFrac, and weighted UniFrac distances, with overall group significance assessed by permutational multivariate analysis of variance (PERMANOVA); pairwise PERMANOVA comparisons were additionally adjusted using the Benjamini–Hochberg FDR procedure. Differentially abundant taxa were identified using Linear Discriminant Analysis Effect Size (LEfSe) [25] with a significance threshold of *p* less than 0.05.

Baseline participant characteristics are reported as mean ± standard deviation for continuous variables and as frequency with percentage for categorical variables. Group differences at baseline were evaluated with independent-samples *t*-tests for continuous measures and chi-square tests for categorical data.

## 3. Results

### 3.1. Characteristics of the Study Participants

Table 1 summarizes the baseline characteristics of participants stratified by MetS status. Among the 24 participants, 54.17% met the criteria for MetS and 45.83% did not. The only statistically significant difference between the two groups was in alcohol consumption: current drinkers were more prevalent in the MetS group, whereas never-drinkers predominated in the control group (*p* < 0.05). All remaining baseline characteristics were comparable between groups.

### 3.2. Taxonomic Composition of Gut Microbiota According to Intake of Dried Laver

**Figure 2:**
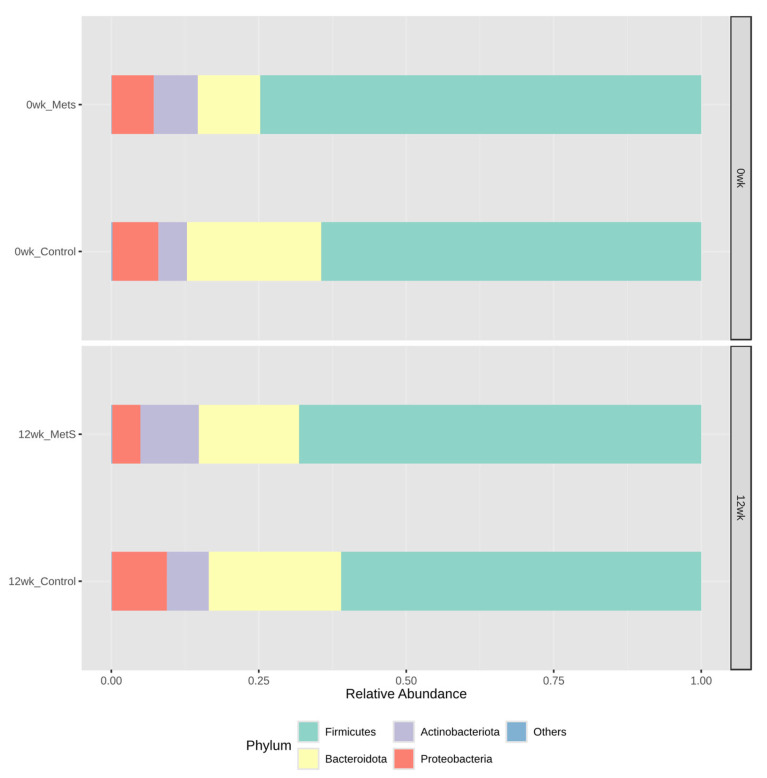
Relative abundance profiles of gut microbiota at the phylum level following 12 weeks of dried laver intake. Low-abundance phyla were grouped into the Others category.

Figure 2 presents phylum-level gut microbiota profiles obtained before and after the 12-week dried laver intervention in both the MetS and control groups. Four phyla were identified in total, with Bacteroidetes and Firmicutes constituting the dominant taxa and collectively representing approximately 90% of the total gut microbiota. Following 12 weeks of laver consumption, the relative abundance of Bacteroidetes increased in the MetS group, and a similar trend toward higher Bacteroidetes proportions was observed in the control group. Relative to baseline, the Firmicutes/Bacteroidetes ratio declined by 0.81-fold in the control group and 0.54-fold in the MetS group, with the reduction being proportionally greater among participants with MetS.

### 3.3. Diversity and Composition of Gut Microbiota According to Dried Laver Intake

**Figure 3:**
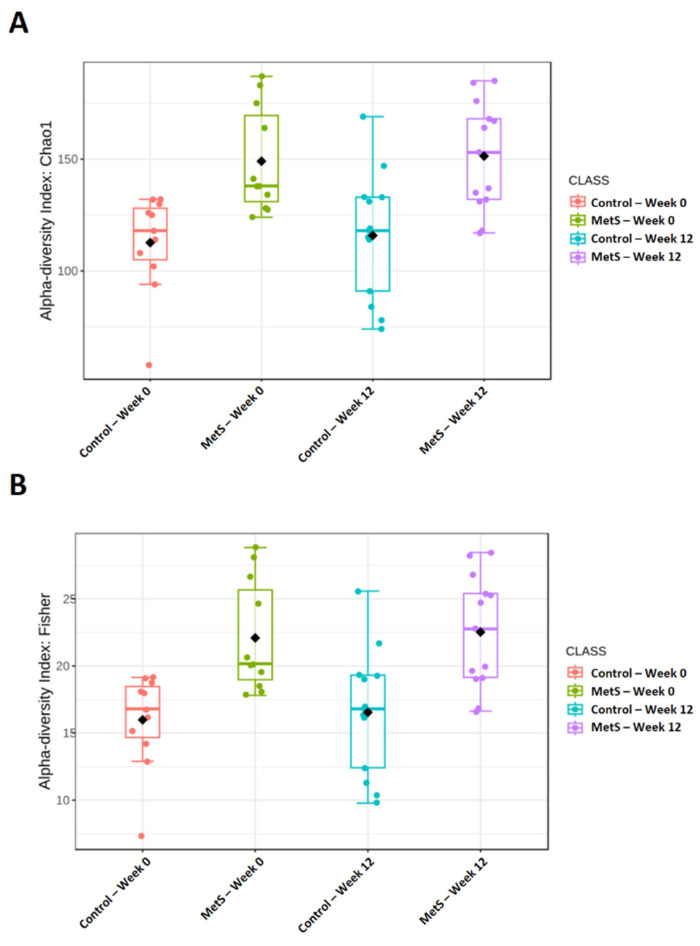
Boxplot of alpha diversity indices according to dried laver intake in the MetS group and control group. (A) Chao1; (B) Fisher. Each colored dot represents an individual sample, with colors indicating the corresponding group and time point. The black diamond indicates the group mean. Group comparisons were performed using Kruskal–Wallis tests followed by FDR-adjusted post hoc pairwise comparisons. MetS, metabolic syndrome.

Alpha diversity was assessed using two complementary indices capturing distinct aspects of community structure. Species richness, quantified by the Chao1 index, differed significantly across groups (Figure 3A; *p* < 0.001), with 12 weeks of dried laver supplementation producing significant increases in both the MetS and control groups. In FDR-adjusted post hoc analyses, significant differences were observed between baseline and week 12 within both groups, whereas no significant between-group differences were detected either at baseline or at week 12. A parallel pattern was observed for the Fisher index (Figure 3B; *p* < 0.001), indicating that the principal alpha-diversity signal reflected temporal change within each group over the intervention period rather than persistent separation according to MetS status.

**Figure 4:**
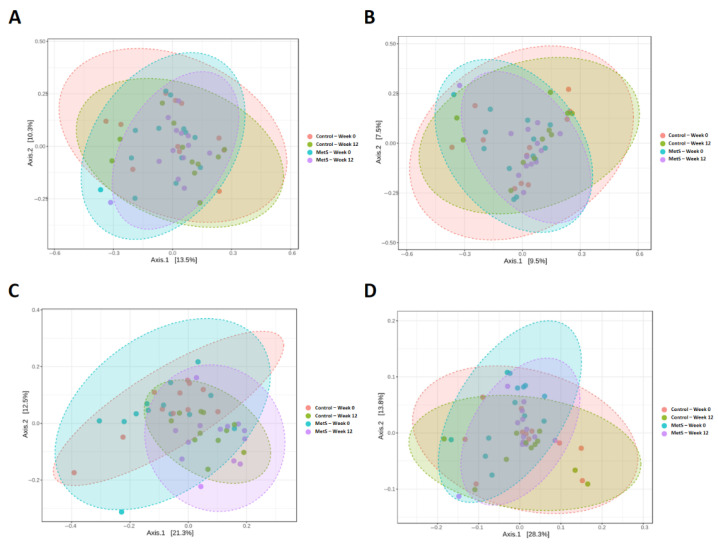
Principal coordinates analysis (PCoA) using beta diversity indices according to dried laver intake in the metabolic syndrome group and control group. (**A**) Bray–Curtis; (**B**) Jaccard; (**C**) unweighted UniFrac; (**D**) weighted UniFrac. Each dot represents an individual sample, and the shaded ellipses indicate the 95% confidence ellipse for each group. Overall group significance was assessed by PERMANOVA, and pairwise PERMANOVA comparisons were adjusted using the Benjamini–Hochberg false discovery rate procedure.

Beta diversity, reflecting the degree of microbial dissimilarity between samples, was evaluated using multiple distance metrics, with community structure visualized by PCoA and between-group significance tested by PERMANOVA. Bray–Curtis dissimilarity revealed significant overall compositional differences across groups (Figure 4A; F = 1.3307, R^2^ = 0.074, *p* = 0.045). However, in pairwise PERMANOVA analyses with FDR correction, no Bray–Curtis comparison remained statistically significant. The Jaccard index, which considers only taxon presence or absence, did not yield significant between-group differences (Figure 4B; *p* > 0.05). Unweighted UniFrac distance, which accounts for phylogenetic relationships based on taxon presence and absence, showed significant group-level differences (Figure 4C; F = 2.4133, R^2^ = 0.141, *p* = 0.001), and FDR-adjusted pairwise analyses demonstrated significant differences between baseline and week 12 within both the MetS group and the control group. Weighted UniFrac distance, which additionally incorporates relative taxon abundances, did not differ significantly between groups (Figure 4D; *p* > 0.05). Collectively, these findings indicate that dried laver intake was associated with shifts in microbial community structure, with the clearest and most consistent signal observed for phylogeny-based presence–absence differences.

### 3.4. Characteristics, Biomarker Features, and Clustering Analysis

**Figure 5:**
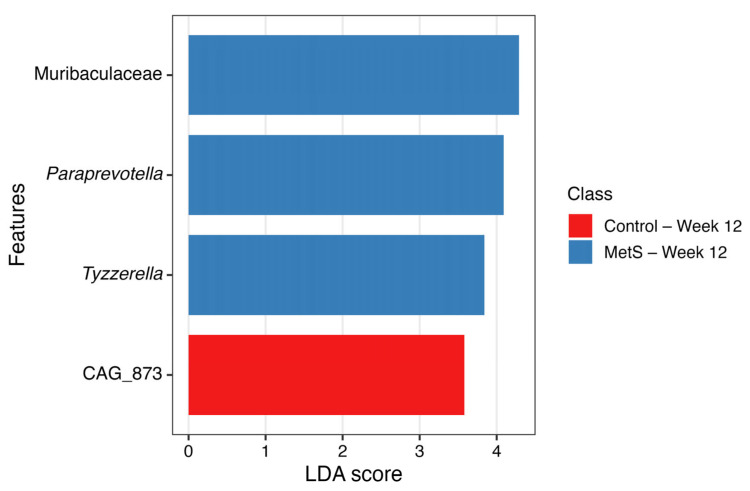
Bar plots of differentially abundant taxa according to intake of dried laver for 12 weeks at genus level. Only features with LDA scores > 3.0 and FDR-adjusted *p* values < 0.05 are displayed. Red bars indicate features enriched in the control group at week 12, and blue bars indicate features enriched in the MetS group at week 12. LDA, linear discriminant analysis; FDR, false discovery rate; MetS, metabolic syndrome.

Differentially abundant taxa following 12 weeks of dried laver consumption were identified using LEfSe analysis with an LDA score threshold of 3.0. At the genus level, four taxonomic features remained significant after FRD adjustment: CAG_873 was enriched in the control group, whereas Muribaculaceae, *Paraprevotella*, and *Tyzzerella* were enriched in the MetS group (all FDR-adjusted *p* < 0.05; Figure 5). 

**Figure 6:**
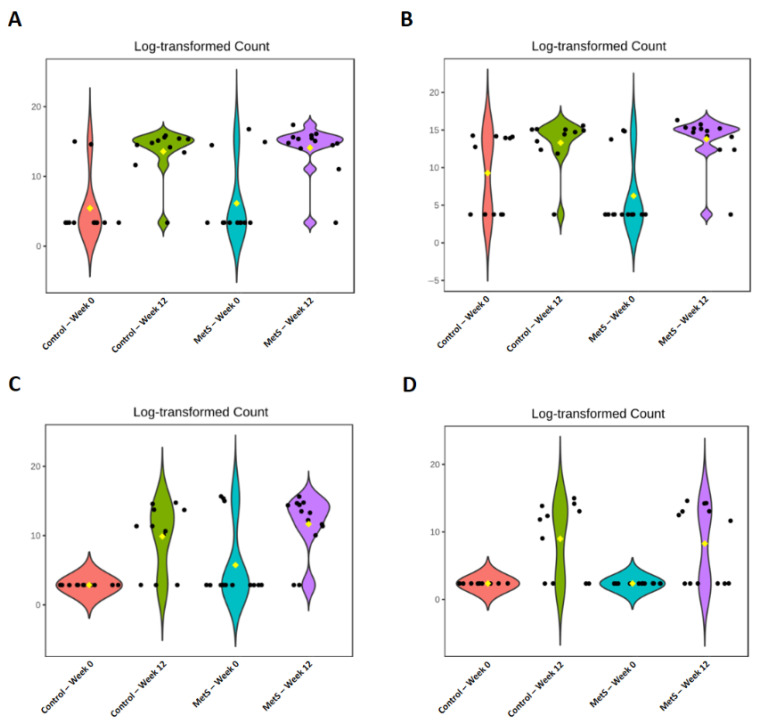
Changes in relative abundance of each microorganism according to dried laver intake in the metabolic syndrome group and control group. (**A**) Muribaculaceae; (**B**) *Paraprevotella*; (**C**) *Tyzzerella*; (**D**) CAG_873. Colors represent the four groups: Control—Week 0 (red), Control—Week 12 (green), MetS—Week 0 (teal), and MetS—Week 12 (purple). The yellow diamond within each violin indicates the mean value. Black dots represent individual data points. MetS, metabolic syndrome.

Violin plots in Figure 6 display the relative abundances of the four FDR-significant genera identified by LEfSe. At baseline, Muribaculaceae was more abundant in the MetS group than in the control group; following 12 weeks of laver intake, abundance increased in both groups, with the MetS group maintaining higher levels throughout (Figure 6A). *Paraprevotella* showed the opposite pattern at baseline, being more prevalent in the control group; after the intervention, both groups exhibited increased abundance, and the MetS group surpassed the control group (Figure 6B). *Tyzzerella* was also more abundant in the MetS group at baseline, and laver consumption elevated its abundance in both groups, with the MetS group retaining higher levels post-intervention (Figure 6C). CAG_873 abundance was comparable between groups at baseline; after 12 weeks, both groups showed increases, with the control group demonstrating a proportionally greater rise (Figure 6D).

## 4. Discussion

To our knowledge, this investigation represents the first clinical study to characterize the effects of dietary dried laver (*Porphyra tenera*) on the gut microbiome in humans, and specifically to examine whether these effects are modified by MetS status. Over 12 weeks of daily supplementation with 4 g of dried *Porphyra tenera*, older women in both the MetS and control groups exhibited measurable shifts in gut microbiome composition and diversity, with some group-specific patterns observed at the taxon level. These findings collectively advance the case for dried laver as a functional food capable of targeting the gut microbiome in a biologically meaningful way.

The Firmicutes-to-Bacteroidetes ratio has long served as a composite index of gut microbiome health, with elevations of this ratio recognized as a hallmark of dysbiosis in obesity and related metabolic conditions [26,27]. Mechanistically, Firmicutes-dominant communities have greater capacity for dietary energy extraction and are associated with heightened intestinal permeability and endotoxemia [28]. At baseline, participants classified with MetS displayed a higher Firmicutes/Bacteroidetes ratio compared with metabolically healthy controls, a pattern consistent with prior MetS microbiome characterizations. After 12 weeks of laver intake, this ratio declined in both groups, with the MetS group exhibiting a proportionally larger reduction. The porphyran content of *Porphyra haitanensis* has previously been shown to reverse an obesity-associated Firmicutes surplus in murine models [19], and the biological mechanisms driving this shift likely reflect the fermentability of laver polysaccharides by Bacteroidetes-affiliated taxa that specialize in sulfated polysaccharide degradation [29]. Although this pattern may suggest that participants with MetS were responsive to the intervention, the present pilot study was not designed to determine whether metabolic status formally modified the magnitude of microbiome response.

Significant changes in alpha diversity following laver supplementation were observed in both groups, as reflected by the Chao1 and Fisher indices. More specifically, FDR-adjusted post hoc analysis indicated significant differences between baseline and week 12 within both the MetS group and the control group, whereas comparisons between the two groups at the same time point were not significant. Reduced microbial diversity—a state associated with diminished functional redundancy and metabolic versatility—is increasingly viewed as a pathological microbiome signature that precedes and accompanies numerous chronic conditions including MetS, inflammatory bowel disease, and type 2 diabetes [30]. Red algal polysaccharides, including carrageenan, agar, porphyran, and xylan, have independently been shown to curb intestinal pathobionts while nurturing commensal beneficial species [29]. Complementary evidence from animal experiments using the green algal polysaccharide ulvan combined with the carotenoid astaxanthin demonstrated salutary effects on both community structure and diversity [31]. Most relevant to the present study, porphyran-derived oligosaccharides from *Porphyra yezoensis* restored microbiome diversity to near-healthy levels in mice with diet-induced non-alcoholic fatty liver disease [32]. Taken together, these findings support the biological plausibility of the alpha-diversity changes observed after dried laver supplementation, while suggesting that the principal signal in the present cohort was temporal change within each group rather than persistent separation according to metabolic status.

Beta diversity analyses provided further evidence that laver consumption was associated with restructuring of gut community composition, although the strength of this signal differed across distance metrics. Bray–Curtis dissimilarity and unweighted UniFrac distance showed significant overall differences, whereas Jaccard and UniFrac did not. However, only unweighted UniFrac retained significant pairwise differences after FDR correction, indicating that the clearest and most consistent intervention-related shift was observed in phylogeny-based presence–absence structure rather than in abundance-weighted community differences. Notably, significant baseline-to-week-12 differences were observed within both the MetS group and the control group, whereas direct comparisons between the two groups at the same time point were not significant. This dissociation between presence–absence and abundance-weighted diversity metrics highlights the nuanced nature of microbiome remodeling in response to dietary intervention and warrants systematic investigation in adequately powered follow-up trials.

Four genera achieved FDR-corrected significance in LEfSe analysis, and their differential patterns illuminate the biological specificity of laver’s prebiotic effects. In control participants, CAG_873 abundance increased markedly following the intervention. This taxon has been linked to fermentation of complex dietary fibers: randomized controlled trial data in overweight men consuming fiber-enriched sorghum documented CAG_873 enrichment alongside improvements in gut microbiome composition and anthropometric outcomes [33], and a similar increase was reported in participants consuming ancient wheat pasta in association with enhanced production of anti-inflammatory SCFAs [34]. The observation that CAG_873 expansion was most pronounced in the non-MetS group may reflect baseline microbiome differences in fiber-fermenting capacity between the two groups. Conversely, published associations between high CAG_873 abundance and hepatic fibrosis [35] and immunoglobulin G decline [36] introduce interpretive uncertainty that warrants dedicated functional investigation before definitive conclusions can be drawn.

The enrichment of Muribaculaceae and *Paraprevotella* in the MetS group following laver intake carries considerable mechanistic significance given their established roles in SCFA biosynthesis. *Paraprevotella*, classified within the phylum Bacteroidetes, is a competent fermentative degrader of dietary fiber polysaccharides, generating acetate and propionate as primary metabolic end-products [37,38]. Muribaculaceae members similarly demonstrate robust propionic acid-producing capacity [39]. SCFAs exert pleiotropic cardiometabolic effects: they suppress luminal pathobionts by lowering colonic pH, reinforce the intestinal mucosal barrier, and modulate innate immune responses [39,40,41,42]. At the systemic level, propionate and acetate signal through G-protein-coupled receptors in adipose, liver, and enteroendocrine tissues to reduce hepatic lipogenesis, enhance insulin sensitivity, and suppress appetite [43,44,45]. The convergence of *Paraprevotella* and Muribaculaceae enrichment with dietary fiber supplementation has been reported in other intervention contexts [46], strengthening the argument that laver-derived polysaccharides selectively cultivate SCFA-producing communities. That these genera were particularly enriched among MetS participants—who presumably entered the trial with a more severely depleted SCFA-producing microbiome—provides an additional dimension to the therapeutic potential of this functional food. However, the classification of these enriched genera as SCFA producers is based on published literature rather than direct measurement in the current cohort. Therefore, SCFA-mediated mechanisms remain a hypothesis that must be tested in future trials incorporating direct fecal and plasma SCFA profiling.

The unexpected rise in *Tyzzerella* abundance in both groups following laver consumption demands careful interpretation. Baseline *Tyzzerella* levels were higher in MetS participants, consistent with published associations between this genus and cardiovascular disease risk and obesity [47,48]. Its continued rise after intervention—despite overall improvements in community composition—was unanticipated. Previous work has identified positive associations between *Tyzzerella* abundance and high intakes of saturated and trans fatty acids [49], yet the current cohort did not receive dietary fat counseling, leaving background diet as a potential but uncontrolled confounder. Cross-feeding ecology—whereby metabolic byproducts generated by laver-enriched taxa secondarily support *Tyzzerella* proliferation—represents a mechanistically plausible but unverified explanation [50]. The net metabolic significance of elevated *Tyzzerella* within an otherwise favorably remodeled community cannot be established from the current study design, and targeted functional metagenomics approaches will be necessary to resolve this question.

The daily supplementation dose of 4 g of dried *Porphyra tenera* used in this study warrants contextualization against habitual laver intake in East Asian populations. In South Korea, mean daily laver (*Porphyra* sp.) intake is estimated at 1.03 g/person/day based on nationally representative survey data, with total seaweed consumption of 3.6 g/day in 2023 [51]. In Japan, nori (*Porphyra* sp.) accounts for approximately 45% of total seaweed consumption, with total dried seaweed intake estimated at 4–7 g/day, suggesting a laver-derived contribution of approximately 1.8–3.2 g/day [52]. In China, where *Porphyra* spp. is among the major edible seaweeds consumed, total seaweed intake has increased from approximately 59 to 94 g/month/person (approximately 2.0–3.1 g/day) between 2004 and 2009, though laver-specific data remain limited [53]. The 4 g/day dose used in the present study therefore modestly exceeds habitual laver-specific intake across all three countries, suggesting that the observed microbiome effects may reflect a supraphysiological rather than strictly habitual exposure. Future dose–response trials using lower, population-representative doses are warranted to determine the minimum effective intake for gut microbiome modulation.

The present findings should be interpreted within the context of several methodological constraints. The study enrolled a demographically homogeneous sample of older Korean women, which, while appropriate for the scientific question, limits generalizability across sexes, ages, and ethnicities. The analytic sample of 24 participants—though sufficient to detect the reported differences—underpowered subgroup analyses and precluded dose–response modelling. The open-label, single-arm design without a concurrent placebo group precludes attribution of microbiome changes solely to laver consumption, and expectation-related behavioral modifications cannot be excluded. Habitual dietary intake was not systematically captured during the intervention period, leaving background diet as an uncontrolled variable. Furthermore, this study did not report changes in clinical metabolic indicators such as waist circumference, blood pressure, and blood glucose, which limits our ability to establish a direct functional link between microbial shifts and host metabolic health. Additionally, alcohol consumption differed significantly between the MetS and control groups at baseline (*p* = 0.0405), which may have acted as a confounding factor in MetS-specific group comparisons. Future randomized, double-blind, placebo-controlled trials should stratify or adjust for alcohol intake, and incorporate comprehensive dietary assessment and shotgun metagenomics to establish causal relationships and identify the specific polysaccharide fractions responsible for the observed effects.

## 5. Conclusions

In this 12-week exploratory pilot study, daily supplementation with 4 g of dried *Porphyra tenera* was associated with increased gut microbial alpha diversity, shifts in beta diversity, a reduction in the Firmicutes/Bacteroidetes ratio, and enrichment of SCFA-producing genera including Muribaculaceae and *Paraprevotella* in older women with MetS. The principal diversity signal reflected temporal change within groups from baseline to week 12, and blood biomarkers were not measured in this study, precluding conclusions about clinical metabolic outcomes. Given the limited sample size and pilot design, these findings should be regarded as preliminary and hypothesis-generating. The potentially greater microbiome responsiveness observed in the MetS group justifies further investigation of dried laver supplementation in populations with pre-existing gut dysbiosis. Adequately powered, randomized, placebo-controlled trials incorporating comprehensive metabolic biomarker assessment are needed before dried laver can be considered as a gut-microbiome-targeted nutritional strategy for MetS prevention or management in older women.

## Figures and Tables

**Table 1:** General characteristics of participants according to presence or absence of metabolic syndrome.

Variables	Metabolic Syndrome (n = 13)	Control (n = 11)	*p* Value
Age (years)	69.15 ± 6.78	63.55 ± 8.17	0.0794
Education			
≤Elementary school	9 (69.23%)	4 (36.36%)	0.2632
Middle school	2 (15.38%)	2 (18.18%)	
High school	2 (15.38%)	3 (27.27%)	
≥College	0 (0.00%)	2 (18.18%)	
Income (won/month)			
<1,000,000	5 (38.46%)	3 (27.27%)	0.4148
1,000,000–3,000,000	5 (38.46%)	3 (27.27%)	
3,000,000–5,000,000	3 (23.08%)	3 (27.27%)	
≥5,000,000	0 (0.00%)	2 (18.18%)	
Drinking status			
Never	4 (30.77%)	8 (72.73%)	0.0405
Current	9 (69.23%)	3 (27.27%)	
Smoking status			
Never	13 (100.00%)	9 (81.82%)	0.1083
Past	0 (0.00%)	2 (18.18%)	
Current	0 (0.00%)	0 (0.0%)	
Marital status			
Single	0 (0.00%)	1 (9.09%)	0.2668
Married	13 (100.00%)	10 (90.91%)	

Continuous variables are presented as mean ± standard deviation (SD), and categorical variables are presented as number (%). The *p*-values were determined using the *t*-test for continuous variables and chi-square test for categorical variables.

## Data Availability

The data supporting the conclusions of this article are not publicly available due to privacy restrictions but may be made available by the authors upon reasonable request.
